# Design and Driving Characteristics of a Bidirectional Micro-Device Based on Multi-Electrothermal Co-Actuation

**DOI:** 10.3390/mi16040487

**Published:** 2025-04-21

**Authors:** Yujuan Tang, Zihao Guo, Yujiao Ding, Xinjie Wang

**Affiliations:** 1 School of Intelligent Science and Control Engineering, Jinling Institute of Technology, Nanjing 211169, China; zhguo020528@163.com; 2 School of Mechanical Engineering, Nanjing University of Science and Technology, Nanjing 210094, China; yujiaoding@njust.edu.cn (Y.D.); xjwang@njust.edu.cn (X.W.)

**Keywords:** multi-electrothermal, V-shaped electrothermal actuator, driving characteristics, finite element simulation

## Abstract

In this paper, a bidirectional micro-device based on multi-electrothermal co-actuation is proposed for a fuze safety system, combining the advantages of the simple structure, small size, low input voltage, large output, and absence of electromagnetic interference in electrothermal actuators. Based on the working principle of the multi-electrothermal co-actuation device and the mathematical model of the single V-shaped electrothermal actuator established in this paper, the temperature distribution of the V-shaped electrothermal actuator is simulated. In addition, the dynamic response and the effect of geometric factors on the output performance of the multi-electrothermal co-actuation device are analyzed in detail. Furthermore, driving characteristics tests of the electrothermal micro-device are carried out. The experimental findings indicate that a displacement of approximately 258.95 μm with a response time of about 156.51 ms can be achieved by the V-shaped electrothermal actuator when the applied voltage is 1.2 V. In a single cycle, a total displacement of 340 μm is obtained by the co-actuation device in around 1.28 s.

## 1. Introduction

With the rapid development of intelligent and miniaturization technology, the electrothermal actuator has gradually become a key driving technology that promotes the innovation and progress of the fuze safety system, possessing great application prospects and high research value. As an important component of the electromechanical system, the micro-actuator converts other forms of energy into mechanical energy and serves as the main device for completing the drive in the mechanism, enabling the output of force or displacement [1]. There are many types of micro-actuators, which are generally categorized based on different driving principles: electrostatic [2], piezoelectric [3], electromagnetic [4], electrothermal [5], and so on. Among them, the electrothermal actuator has become one of the important driving methods in the micro-actuator field due to its simpler structure, smaller size, lack of electrostatic or magnetic field interference, and better compatibility with integrated circuits [6].

Electrothermal actuators are classified into three types based on their deformation modes after heating: coplanar [6], out-of-plane [7], and three-dimensional actuators [8]. Since the isolation device in the fuze safety system primarily adopts a stacked sheet assembly method, its deformation remains mainly confined within the same plane. Therefore, choosing a coplanar structure actuator is most appropriate, and the V-shaped structure is the most widely used. Many studies have investigated the performance and applications of electrothermal actuators.

Shen et al. [9] investigated the differences between single and cascaded V-shaped electrothermal actuators. The maximum deflection of the former occurs at a 0.04 rad beam angle, whereas the latter reaches a beam angle of 0.19 rad. Additionally, the deflection remains independent of the beam thickness, providing a foundation for the structural optimization of V-shaped electrothermal actuators. A novel V-shaped electrothermal actuator fabricated by Shen et al. [10] using surface silicon technology achieved significant displacement at lower voltages within a shorter response time. Nguyen et al. [11] developed an electrothermal V-shaped actuator using a surface sputtering process. Under a voltage of 16 V, the displacement of the sputtering V-shaped actuator was twice that of the non-sputtering one, enhancing the displacement output capability of the electrothermal actuators under the same voltage conditions. Cao et al. [12] conducted an in-depth study on electrothermal actuators’ heat transfer mechanisms and dynamic behavior in MEMS applications. An electrothermal–structural coupling model was established, providing new insights into the characteristic analysis of electrothermal actuators under multi-physics field coupling. Tecpoyotl et al. [13] designed a novel asymmetric Z-shaped electrothermal actuator. At a voltage of 2 V, the output force increased by 370.48%, while the displacement decreased by 29.8%, making it suitable for applications where a large driving force is required but the displacement output is not a critical factor. Vargas et al. [14] studied an electrothermal actuator based on a rotary system. The claw displacement of the actuator reached 21.2 μm, with a driving force of approximately 34.2 μN. Although this actuator offers significant advantages in terms of miniaturization, the array-based layout of its driving beams results in a complex structure. Hoang et al. [15] employed a PSO algorithm to optimize a V-shaped actuator. At a maximum voltage of 28 V, the optimized design provided a displacement approximately 22% larger than that of the non-optimized design while ensuring compliance with safe operating conditions.

As mentioned above, electrothermal actuators exhibit unidirectional displacement output, and their displacement per single electrical signal is smaller than their size. Therefore, in recent years, researchers have explored novel structures and cooperative actuation techniques to achieve bidirectional larger displacement. Hu et al. [16] designed a bidirectional large-displacement MEMS platform consisting of multiple electrothermal actuators and used a claw-type actuator design to achieve large displacement. The symmetrical arrangement of the structure enables bidirectional actuation. However, the device’s varying configurations and many electrothermal actuators result in a complex structure. Additionally, the micro-lever features two sharp corners, making it less suitable for laser processing. To address these issues, this study proposes a bidirectional displacement device based on multi-electrothermal actuator co-actuation. The structure of the device adopts a symmetrical configuration, reducing the number of electrothermal actuators to four. Moreover, the micro-lever is redesigned as a straight lever, making it more suitable for laser processing.

## 2. Working Principle of the Multi-Electrothermal Co-Actuation Device

**Figure 1:**
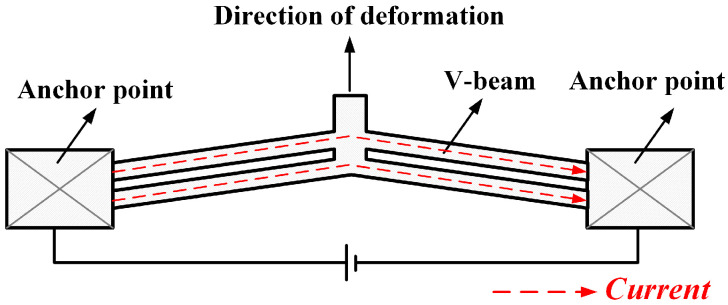
The structure diagram of the V-shaped electrothermal actuator.

The electrothermal actuator mainly uses the material’s thermal expansion effect. The V-shaped electrothermal actuator consists of two end anchor points and a V-shaped actuate beam [17]. The structure is shown schematically in Figure 1. When a voltage is applied at both ends of the anchor point, current flows along the V-beam, generating Joule heat through the beam’s internal structure, causing the actuator to expand thermally.

**Figure 2:**
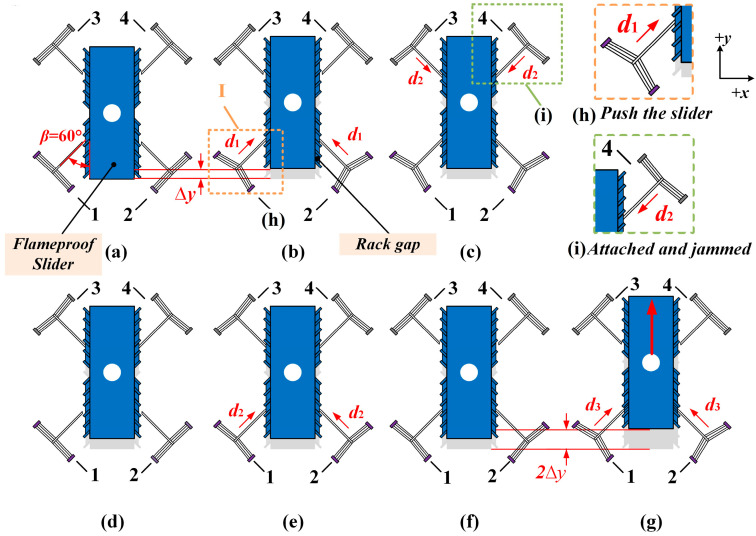
The working process of the multi-electrothermal co-actuation device. (**a**) Initial state; (**b**) actuators 1 and 2 push the slider to move one sub-step displacement; (**c**) actuator 3 and actuator 4 fix the position of the slider; (**d**) the levers of actuators 1 and 2 return to the initial position; (**e**) the levers of actuators 1 and 2 only contact the slider; (**f**) actuators 3 and 4 are powered off and move back to the initial position; (**g**) actuators 1 and 2 push the flameproof slider again to output another sub-step displacement; (**h**) the detail of actuators 1 and 2 pushing the slider; and (**i**) the detail of actuators 3 and 4 jamming the slider.

Typically, the displacement of a single electrothermal actuator is several tens of microns. This makes it difficult to meet the requirement for the large displacement of the flameproof slider in the state transition of the fuze safety system. In this paper, the V-shaped multiple electrothermal actuators co-actuate to realize the large displacement in the bi-direction of the flameproof slider in the fuze system. As illustrated in Figure 2, when the flameproof slider is required to output displacement in the +*y* direction, it is mainly actuated by actuators 1 and 2. Conversely, when the slider needs to output displacement in the −*y* direction, the predominant actuators are actuators 3 and 4. The device’s working process in the +*y* direction within one cycle time can be subdivided into six steps. Figure 2a shows the device’s initial state.

1. Step 1: Voltage U1 applies to actuators 1 and 2 simultaneously and produces a deformation of *d*_1_. The levers of actuators 1 and 2 push the slider to move one sub-step displacement Δ*y*, as shown in Figure 2b. The detail can be seen in Figure 2h.
2. Step 2: Actuators 1 and 2 are still powered on. Voltage U2 (U2 < U1) applies to actuators 3 and 4 at the same time. It produces a deformation of *d*_2_; the levers of actuators 3 and 4 only contact the outer wall of the slider, which is not enough to push the slider to move. Actuator 3 and actuator 4 just play a role in fixing the position of the slider, as shown in Figure 2c. The detail can be shown in Figure 2i.
3. Step 3: While actuator 3 and actuator 4 remain continuously powered on to fix the slider, actuators 1 and 2 are powered off at the same time, and the levers of actuators 1 and 2 will separate from the slider and return to the initial position, as shown in Figure 2d.
4. Step 4: Actuator 3 and actuator 4 remain energized, and at the same time, the voltage U2 is applied to actuator 1 and actuator 2, causing thermal deformation of *d*_2_ so that the levers of actuators 1 and 2 only contact the slider but will not push the slider to move, as shown in Figure 2e.
5. Step 5: With actuators 1 and 2 remaining powered on, actuators 3 and 4 are powered off simultaneously and move back to the initial position, as shown in Figure 2f.
6. Step 6: At the same time, the voltage applied to actuators 1 and 2 will increase from U2 to U1, and the temperature load will increase. Finally, actuators 1 and 2 will produce a deformation of *d*_3_ (*d*_1_ ≈ *d*_2_ + *d*_3_) to push the flameproof slider again to output another sub-step displacement Δ*y*, as shown in Figure 2g.

At the end of one cycle time, the total displacement of the slider is 2Δ*y*. The direction of movement of the slider depends on the position of the detonating hole of the fuze mechanism, and the conversion between the safety state and the pending state in the fuze is achieved by aligning and staggering the flameproof hole and the detonating hole. Assume that the detonation hole is located above the flameproof slider, so the flameproof slider must move in +*y* direction; at this time, actuator 1 and actuator 2 can be regarded as the main-actuate unit responsible for pushing the slider output displacement, while actuators 3 and 4, as the sub-actuate unit, play a position-holding role. Similarly, suppose the detonation hole is below the flameproof slider. In that case, the slider must move in the −*y* direction, and the working process is the same as in Figure 2, except that the working order of actuators 1 and 2 and actuators 3 and 4 is reversed.

In addition, the operating conditions are the same for each movement step, so the total displacement of the co-actuation device increases with the increase in the cycle numbers and depends only on the slider’s rack gap size.

## 3. Theory and Simulation Analysis of the V-Shaped Electrothermal Actuator

This paper focuses on the study of metal-based electrothermal actuators. Since different metals have different heat resistance, the temperature characteristics of the actuator will vary due to the selection of materials. In this study, stainless steel 304 is finally selected as the main material for the V-shaped electrothermal actuator.

### 3.1. Mathematical Model of the Single V-Shaped Electrothermal Actuator

**Figure 3:**
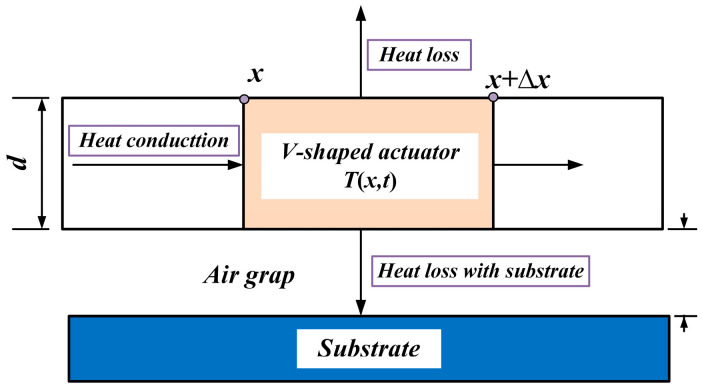
The heat condition path in the unit length of the V-shaped beam.

Heat conduction, convection, and radiation are the three forms of heat transfer. For micro-devices, the effects of heat convection and radiation on their performance are relatively small. Therefore, in the theoretical model, the heat radiation and convection effects are neglected. The heat conduction path of the V-shaped electrothermal actuator is approximately shown in Figure 3.

The temperature distribution of the actuator is simplified to a one-dimensional steady-state problem [18]. By combining the principle of energy conservation, a transient mathematical model for the V-shaped electrothermal actuator can be derived as follows:

(1) $$C_{p}\rho \frac{\partial T_{r}}{\partial t}=\kappa \left(T_{r}\right)\frac{\partial^{2}T_{r}}{\partial x^{2}}-\left[\frac{S_{a}\left(a+2d\right)}{ad}+\frac{S_{t}}{d}\right]\left(T_{r}-T_{0}\right)+J^{2}\lambda \left(T_{r}\right)\begin{array}{cc} & r=\left\{Z,Y\right\} \end{array}$$

where *C_p_* and *ρ* are the constant pressure-specific heat capacity and density of the material.

**Figure 4:**
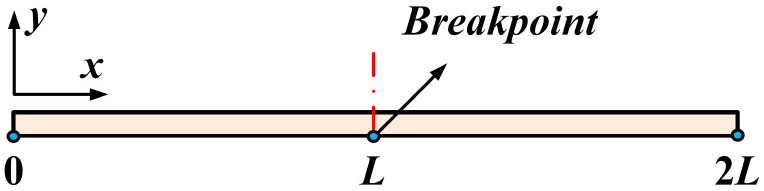
The one-dimensional simplified model of the V-shaped beam.

The V-shaped electrothermal actuator’s beam width and thickness are much smaller than its beam length; therefore, it can be simplified as a one-dimensional structure, as shown in Figure 4.

The simplified model shown in Figure 4 indicates a breakpoint between the intervals [0, *L*] and [*L*, 2*L*], corresponding to the electrothermal actuator’s center point. The temperature distribution function at this discontinuity is second-order derivable, which leads to the relationship given in:

(2) $$\left\{\begin{array}{l} T_{Z}\left(x_{0},t\right)=T_{Y}\left(x_{0},t\right)\begin{array}{cc} & x_{0}=L \end{array}\\ \kappa \left(T_{Z}\right){\left[\frac{\partial T}{\partial x}\right]}_{x=x_{0}}=\kappa \left(T_{Y}\right){\left[\frac{\partial T}{\partial x}\right]}_{x=x_{0}} \end{array}\right.$$

At the initial time *t* = 0, the temperature of each region of the electrothermal actuator is assumed to be equal to the room temperature *T*_0_, and the system is in a steady state. During operation, most of the energy at the anchor points is transferred to the internal structure of the V-beam or dissipated in the form of heat conduction. Therefore, it can be approximated that the temperatures near the anchor points of the V-shaped actuator remain at *T*_0_. The boundary conditions of the solution can be given as follows:

(3) $$\left\{\begin{array}{l} T_{r}\left(x,0\right)=T_{0}\begin{array}{cc} & r=\left\{Z,Y\right\} \end{array}\\ T_{Z}\left(x_{0},t\right)=T_{0}\begin{array}{cc} & x_{0}=0 \end{array}\\ T_{Y}\left(x_{0},t\right)=T_{0}\begin{array}{cc} & x_{0}=2L \end{array} \end{array}\right.$$

**Figure 5:**
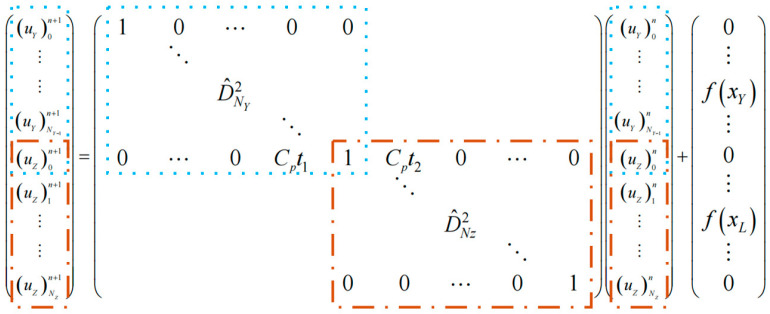
The solution matrix equation in the transient state of the temperature distribution.

Equation (1) is temporally and spatially discretized using the Chebyshev spectral method [19] and the forward Euler difference scheme [20]. Figure 5 shows the resulting matrix equation for solving the transient temperature distribution of the V-shaped electrothermal actuator.

Since the displacements resulting from the thermal deformation of micro-devices are in the order of nanometers or micrometers, they are difficult to describe directly using the traditional macroscopic motion equations. Therefore, the actuator can be modeled as a discrete vibration system [21], and the forced vibration equation can be used to characterize the transient motion in the central region of the V-shaped electrothermal actuator. By combining the temperature distribution equation, the theoretical displacement of the V-shaped electrothermal actuator can ultimately be obtained:

(4) $$y=\sqrt[{n+1}]{\frac{F_{z}^{n}\Delta t^{2}+2m_{z}y^{n}-m_{z}y^{n-1}}{m_{z}+\Delta t^{2}\cdot {K_{z}}^{n}}}$$

where the parameters in Equation (4) are represented as follows:

(5) $$\left\{\begin{array}{l} K_{z}^{n}=\frac{2EAh\theta^{n}}{L\cdot l}+\frac{6EI\cos\theta^{n}}{l^{3}}\\ \theta^{n}=\frac{\arctan\left(l\sin\theta_{0}+y^{n}\right)}{l\cos\theta_{0}}\\ F_{z}^{n}=\frac{2\Delta lEA}{l\cdot \sin\theta^{n}} \end{array}\right.$$

An electrothermal actuator’s dynamic response time refers to when the actuator receives an electrical signal and produces an observable mechanical response (e.g., displacement). According to the working principle of the electrothermal actuator, there is a thermal response [22] to the electrical–thermal generation of Joule heat and a mechanical response to the thermal-force-generated deformation and, thus, output displacement. Therefore, the electrothermal actuator can be viewed as a first-order thermal response system in series with a second-order mechanical response system [23]. Since the thermal response time is typically longer than the mechanical response time, the latter can be neglected in the theoretical analysis. The transfer function of the electrothermal actuator can be expressed as follows:

(6) $$G\left(s\right)=G_{T}\left(s\right)=\frac{1}{\tau s+1}$$

where *τ* is the time constant, generally referring to the time required for the system to reach 63.2% of its steady-state value [24].

When the electrothermal actuator is suddenly powered on, its thermal response will rise with time and eventually reach a stable temperature. Therefore, the input can be considered a unit step function. After applying the Laplace inverse transform, the time domain response curve of the actuator can be obtained:

(7) $$y\left(t\right)=1-e^{-\frac{t}{\tau }}$$

The response time is typically defined as the time required for the system output to reach 95% or 98% of its steady-state value, referring to the rise time. For first-order systems, it can be approximated as 3τ or 4τ [25].

### 3.2. Temperature Distribution and Displacement Simulation of the V-Shaped Electrothermal Actuator

**Figure 6:**
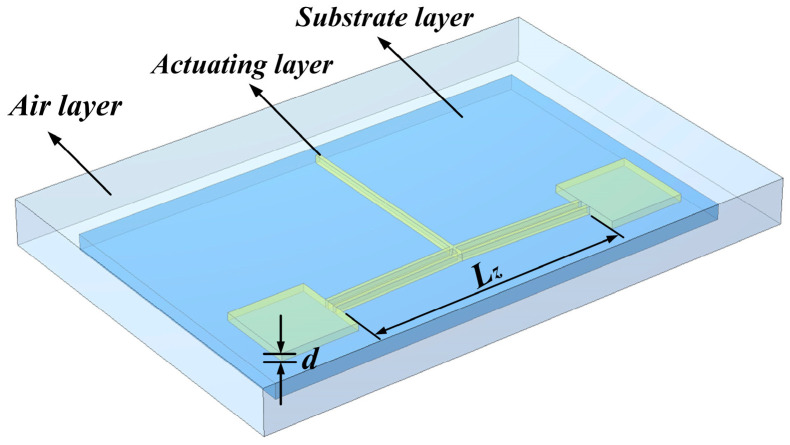
The model of the V-shaped actuator for the finite element analysis.

The finite element 3D model of the V-shaped electrothermal actuator, shown in Figure 6, is established, and its output characteristics are analyzed using finite element software.

**Figure 7:**
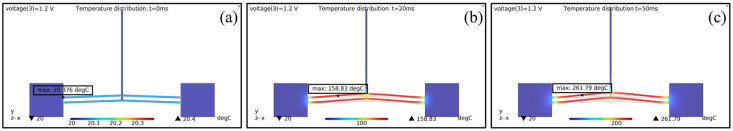
Temperature distribution of the V-shaped actuator at different times with 1.2 V voltage: (**a**) initial state; (**b**) t = 20 ms; and (**c**) t = 50 ms.

Figure 7 shows the diagram of the temperature distribution of the V-shaped actuator at the initial moments, 20 ms and 50 ms, when the driving voltage is 1.2 V under simulation analysis. At each moment, the highest temperature is observed at the V-beam arms of the actuator rather than at the center of the lever, indicating that the V-beams of the metal-based electrothermal actuator are the main areas of heat concentration.

The temperature distribution on the V-beam exhibits good symmetry, which indicates that the heat transfer is uniform and stable, with the temperature being evenly distributed on the V-beam. Furthermore, the maximum temperature gradually increases with the time, and the degree of deformation caused by the thermal effects becomes more pronounced, proving that the thermal effect is a dynamic process that accumulates with time.

**Figure 8:**
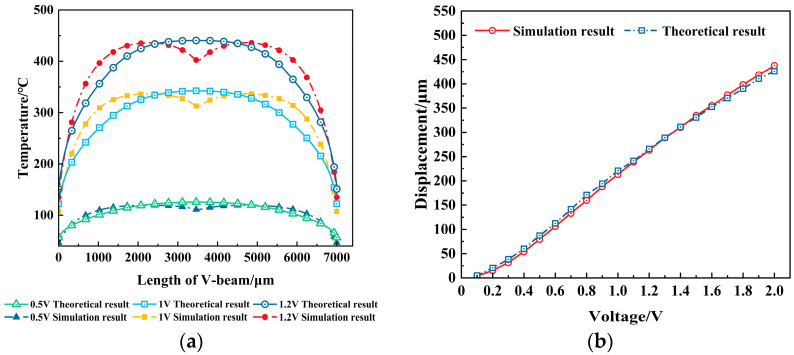
Temperature distribution and displacement simulation results: (**a**) temperature distribution at different voltages in a steady state; and (**b**) displacement at different voltages at a steady state.

Figure 8a displays the electrothermal actuator’s theory and simulation temperature distribution in the steady state under different voltages. The theory temperature distribution curve is approximately a quadratic parabolic curve, while the simulation results exhibit an “M”-shaped temperature distribution curve; there is some error between them. As the voltage gets lower and closer to the steady state, the error is smaller and the temperature distribution is more uniform.

Figure 8b shows the variation curve of the steady-state displacement of the V-shaped actuator with the voltage, which visually reflects the relationship between voltage and displacement under theoretical and simulation analysis. The output displacement shows a nonlinear growth trend with the increase in the voltage.

**Figure 9:**
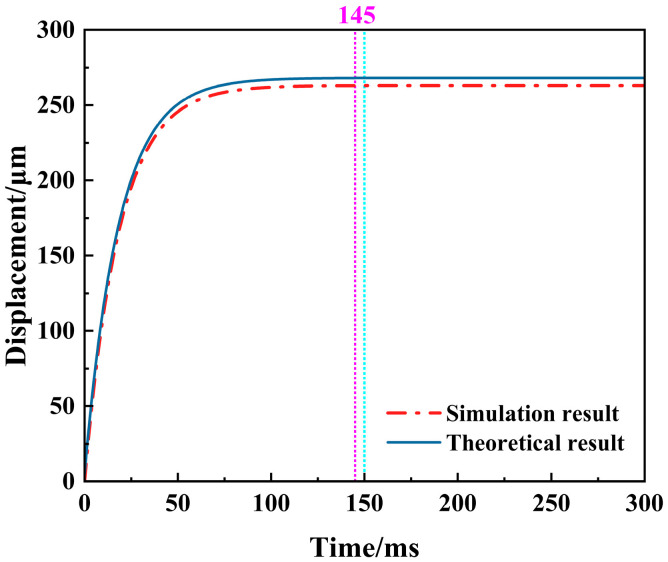
Dynamic response curve of the V-shaped electrothermal actuator at 1.2 V voltage.

Figure 9 shows the dynamic response curve under the theoretical and simulation results of the V-shaped actuator at 1.2 V voltage. The curve’s trend shows that the actuator’s output displacement increases rapidly with time. After a period of time, the displacement no longer changes, indicating that the actuator has reached a steady state.

The response time of the V-shaped electrothermal actuator under the simulation results is about 150 ms, and the response time under the theoretical results is approximately 145 ms, with the theoretical value being slightly lower than the simulation value. This discrepancy arises because the mechanical response time is ignored in the theoretical analysis and the thermal response time is directly taken as the overall system response time. The error between them is about 3.45%, which is within the allowable error range.

## 4. Dynamic Response Characteristic of the Multi-Electrothermal Co-Actuation Device

The system response time of the multi-electrothermal co-actuation device is mainly determined by the driving strategy and response characteristics of the V-shaped electrothermal actuators.

**Figure 10:**
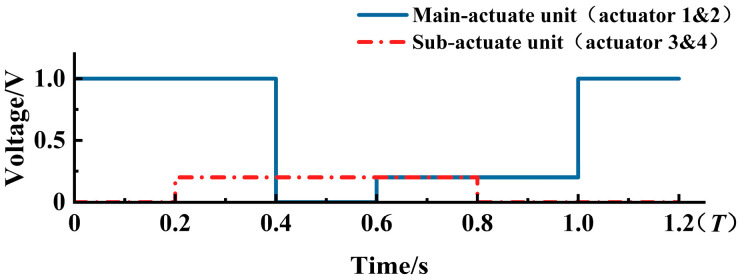
The voltage pulse of each actuator within one cycle time.

Based on the performance analysis of a single V-shaped actuator in Section 3.2, the voltage for the main-actuate unit (actuators 1 and 2) is set to U1 = 1.0 V, while the voltage of the sub-actuate unit (actuators 3 and 4) is given as U2 = 0.2 V. One working time step is set to 200 ms, where the voltage pulse within one cycle of each actuator is obtained, as shown in Figure 10.

**Figure 11:**
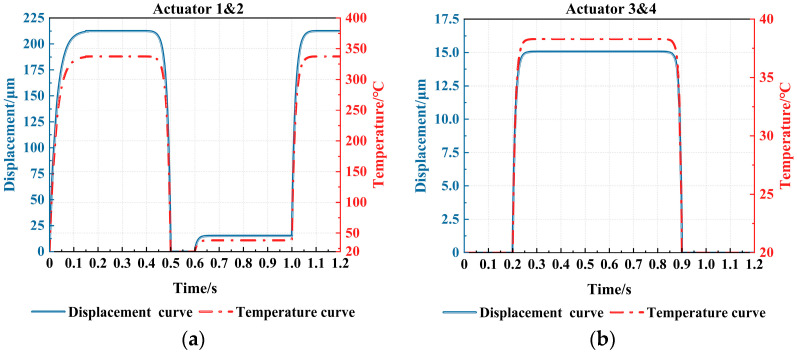
The displacement and temperature curve of the actuators: (**a**) the main-actuate unit; and (**b**) the sub-actuate unit.

As shown in Figure 11a,b, the dynamic response curves of the main-actuate unit and the sub-actuate unit within one cycle are, respectively, illustrated. Throughout the process, actuators 1 and 2 in the main-actuate unit are primarily responsible for driving the flameproof slider, which can be actuated twice in a single cycle, with a maximum displacement of about 213 μm and a maximum temperature of about 340 °C. The actuators 3 and 4 in the sub-actuate unit have a lower input voltage, the maximum temperature is approximately 40 °C in each cycle, and the maximum displacement is about 15.08 μm, which ensures the effective engagement between the levers of actuators 3 and 4 and the rack gap of the flameproof slider, thus achieving the position-holding function.

**Figure 12:**
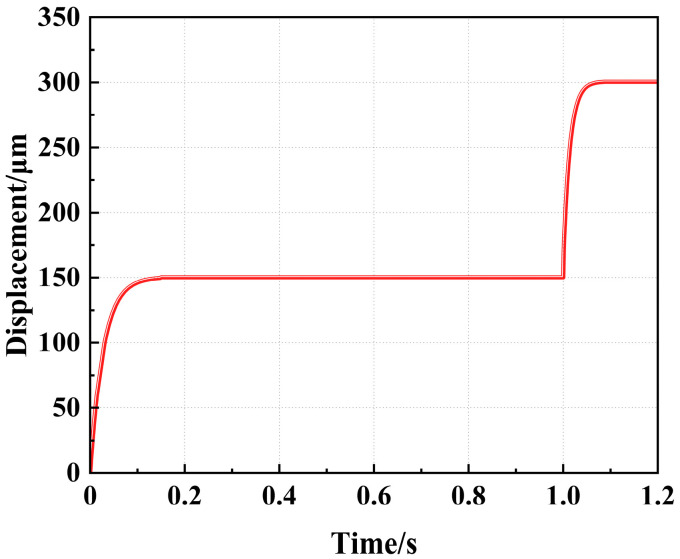
The dynamic response of the flameproof slider.

Figure 12 presents the flameproof slider’s dynamic response curve within one cycle. The slider can cumulatively output two steps within one cycle, its total displacement is about 300.14 μm, and its overall system response time is 1.2 s.

## 5. The Effect of Geometric Factors on the Output Performance of the V-Shaped Actuator

The performance of the device in the fuze directly determines the reliability of the system’s operation. The dimensional parameters of the electrothermal actuator and the surrounding environment significantly affect the performance of the fuze device, and it is crucial to analyze in depth the effect of various geometric factors on its performance.

**Figure 13:**
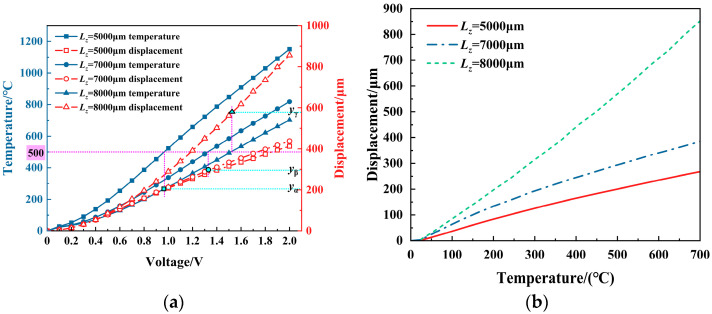
The effect of the V-beam length (*L_z_*) on the displacement of the V-shaped electrothermal actuator: (**a**) under different voltages; and (**b**) under different temperatures.

Figure 13 shows the simulation results of the output displacement under different V-beam lengths *L_z_*. The results indicate that the output displacement increases with the length for the same voltage and temperature situations. Moreover, when the length is longer (e.g., *L_z_* = 8000 μm), the displacement variation with the length becomes more pronounced.

Therefore, when selecting or optimizing the V-beam length, a larger value should be chosen as much as possible while meeting the requirements of the dimensional constraints.

**Figure 14:**
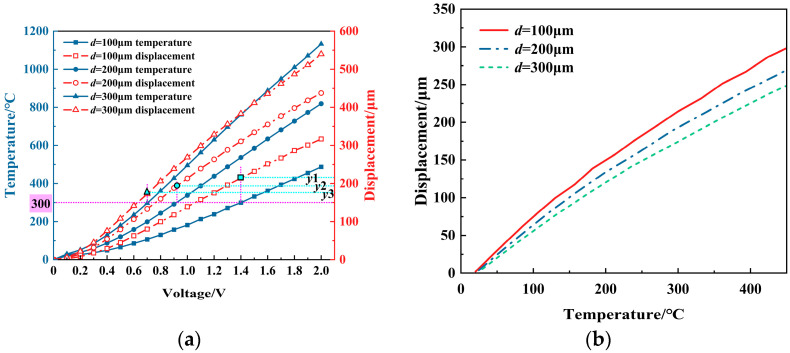
The effect of the V-beam thinness (*d*) on the displacement of the V-shaped electrothermal actuator: (**a**) under different voltages; and (**b**) under different temperatures.

Figure 14 presents the results of the effect of the V-beam thickness on the output displacement. The output displacement increases with the increase in thickness under the same voltage. However, for the same temperature, a larger thickness results in a smaller corresponding displacement. This is because, under the same temperature load Δ*T*, an increase in thickness raises the structural stiffness of the actuator, thus limiting the displacement output.

Overall, the V-beam thickness should be as thin as possible, both to allow for easier access to sheet material and to ensure structural reliability and performance requirements are met.

**Figure 15:**
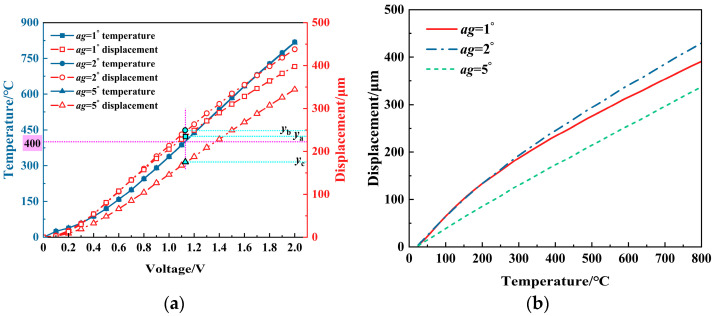
The effect of the title angle (*ag*) on the displacement of the V-shaped electrothermal actuator: (**a**) under different voltages; and (**b**) under different temperatures.

Figure 15 shows the results of the effect of the tilt angle on the displacement. The analysis reveals that, under the same voltage and temperature, the displacement increases and then decreases with the increase in the tilt angle. When the tilt angle is within a smaller range (e.g., 1° to 2°), its variation has less effect on the displacement.

**Figure 16:**
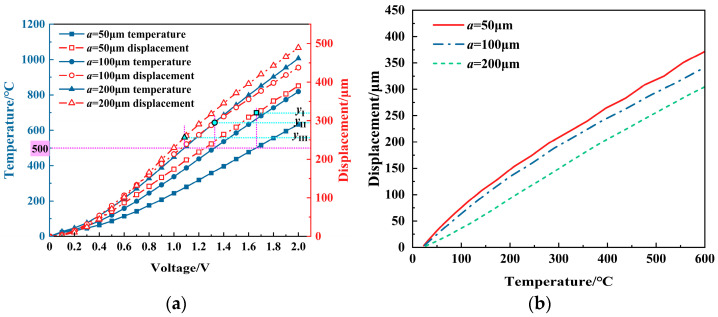
The effect of the V-beam width (*a*) on the displacement of the V-shaped electrothermal actuator: (**a**) under different voltages; and (**b**) under different temperatures.

Figure 16 shows the impact of the V-beam width on the displacement. Under the same voltage condition, the displacement shows a nonlinear increasing trend with the increase in the width, while under the same temperature condition, the displacement decreases with the increase in the width. The increase in width promotes the rigidity of the structure, which inhibits the displacement under the same temperature load. Nevertheless, within the range of width, the variation in its value has relatively less effect on the displacement.

Therefore, when determining the geometric parameters of the V-shaped actuator, an appropriate V-beam width should be selected. The value should not be too large to affect the output performance or too small to ensure processing accuracy.

Comprehensively, when analyzing from the perspective of the same voltage conditions, the V-beam thickness and length are sensitive factors, while the width and tilt angle are insensitive factors. When analyzing from the point of view of the same temperature load, the length is the sensitive factor, while the thickness, beam width, and tilt angle are insensitive factors. Therefore, in the design phase, if it is impossible to select the optimal values for all the geometric parameters due to some limitations, the sensitive geometric factors can be prioritized for selecting the optimal values. Then, the insensitive geometric factors can be selected.

According to the requirements of modern fuze miniaturization in terms of the overall dimensions of the mechanism, to optimize the output performance of the multi-electrothermal co-actuation device, the final geometric parameters are provided in Table 1.

The experimental prototype will be fabricated in Section 6 based on the parameters in Table 1.

## 6. Driving Characteristics Test of the Electrothermal Micro-Device

**Figure 17:**
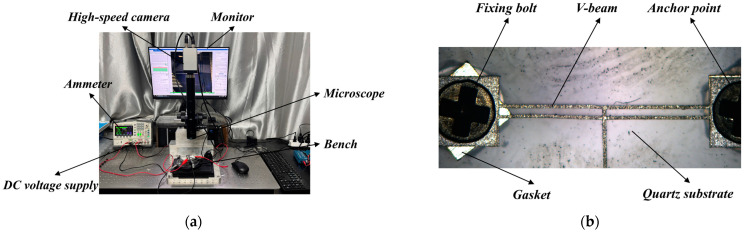
The experimental preparation of the electrothermal micro-device: (**a**) the experimental platform; and (**b**) the prototype of the V-shaped electrothermal actuator.

The output displacement and response time are important indicators for measuring the driving performance of electrothermal actuators. The performance experimental platform for the electrothermal actuator consists of a microscope, DC voltage supply, ammeter, high-speed camera with monitor, and measurement software, as shown in Figure 17a.

After fabricating the actuator prototype by laser processing, the anchor points at both ends are bolted to the substrate to ensure its stability during the experiment, as shown in Figure 17b.

### 6.1. Output Performance Test of the V-Shaped Electrothermal Actuator

In this experiment, different voltages will be applied to the V-shaped electrothermal actuator prototype to observe its steady-state and transient displacements under different voltages as well as the dynamic response time.

#### 6.1.1. Displacement and Temperature Distribution Test in a Steady State

**Figure 18:**
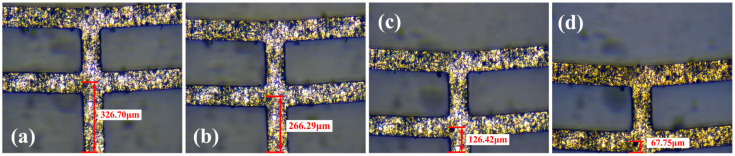
The deformation of the V-shaped actuator in a steady state under different voltages: (**a**) initial state; (**b**) 0.5 voltage; (**c**) 1.0 voltage; and (**d**) 1.2 voltage.

For the performance of the electrothermal actuator in a steady state, the test method mainly evaluates the output displacement and power, and it performs comparisons with the simulation analysis results for verification. The displacement at each voltage is obtained by measuring the distance from the reference point on the electrothermal actuator to the reference line (which is not changed with the movement of the actuator, i.e., the lower boundary line of the field of view), and subtracting the distance in the initial state, the final experimental results are shown in Figure 18.

**Figure 19:**
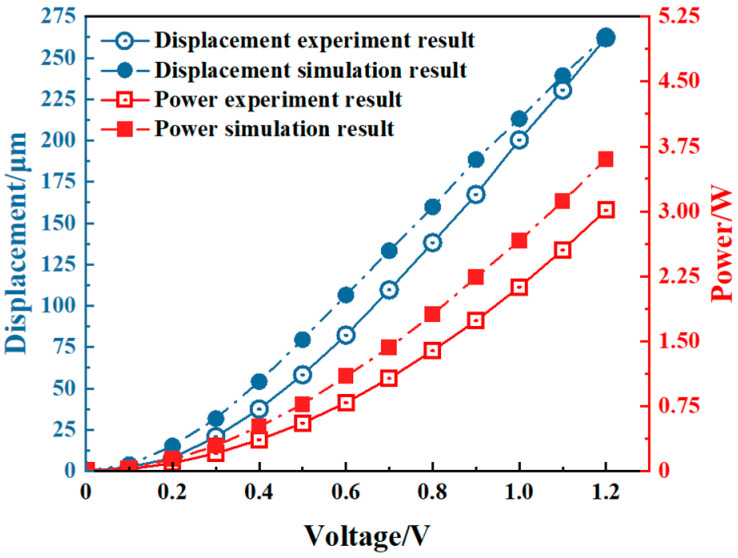
The simulation and experiment results of the V-shaped electrothermal actuator.

Figure 19 shows the comparison curves between the experimental results and the simulation results. The results indicate that both the displacement and the power increase nonlinearly with the voltage. The experimental results are basically consistent with the trend observed under simulation. The experimental values are slightly smaller than the simulation values, and the error is within the allowable range.

**Figure 20:**
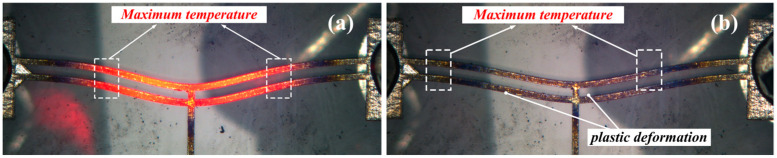
The temperature distribution of the V-shaped electrothermal actuator: (**a**) the temperature distribution under 2.0 V voltage; and (**b**) the status of the actuator after a period of power off.

As shown in Figure 20a, when a 2.0 V voltage is applied to the electrothermal actuator, the highest temperature occurs in the V-shaped actuator beam arm, which is completely consistent with the simulation results in Section 3.2, thereby validating the accuracy of the electrothermal actuator’s temperature distribution theoretical model.

As shown in Figure 20b, when the input voltage is higher (resulting in a higher temperature), irreversible oxidation may occur on the material surface of the driving beam. Even after disconnecting the power supply, plastic deformation still exists in the V-beam once the material’s temperature exceeds its yield point. The high-temperature regions appear deep blue, while the low-temperature regions appear orange, demonstrating the temperature distribution differences in the driving beam.

#### 6.1.2. Dynamic Characterization Test

**Figure 21:**
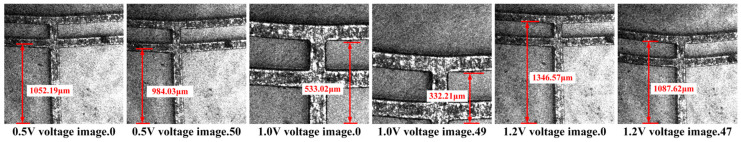
The temperature distribution of the V-shaped electrothermal actuator.

This experiment uses a high-speed camera to record the actuator’s deformation process under voltages of 0.5 V, 1.0 V, and 1.2 V. The results are shown in Figure 21.

The high-speed camera’s frame rate is 300 frames per second. By calculating the number of frames, the dynamic response time experimental results for the electrothermal actuator at different voltages are 166.5 ms (0.5 V), 163.17 ms (1.0V), and 156.51 ms (1.2 V), respectively. When reaching the steady state, the displacements are 68.16 μm (0.5 V), 200.81 μm (1.0 V), and 258.95 μm (1.2 V), which are in good agreement with those measured under the steady state.

**Figure 22:**
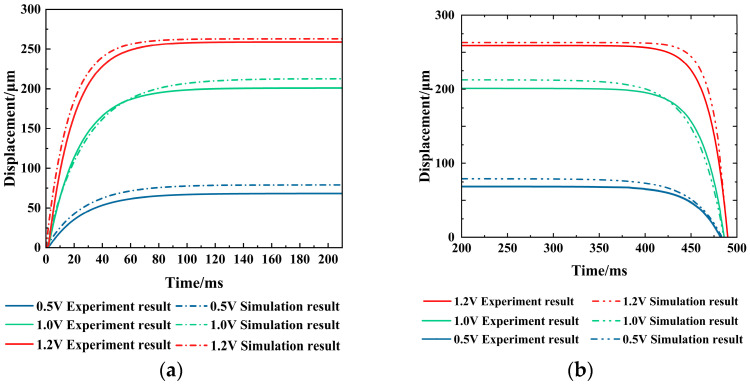
The dynamic response of the V-shaped electrothermal actuator under different voltages: (**a**) the dynamic of rising to a steady state; and (**b**) the dynamics of returning to the initial state.

Figure 22 compares the experimental and simulated dynamic responses of the electrothermal actuator with voltages of 0.5 V, 1.0 V, and 1.2 V. The experimental results are basically consistent with the simulation results.

### 6.2. Driving Characteristics of the Multi-Electrothermal Co-Actuation Device

The total cumulative displacement within one cycle is an important indicator for assessing the displacement performance of the co-actuation device.

**Figure 23:**
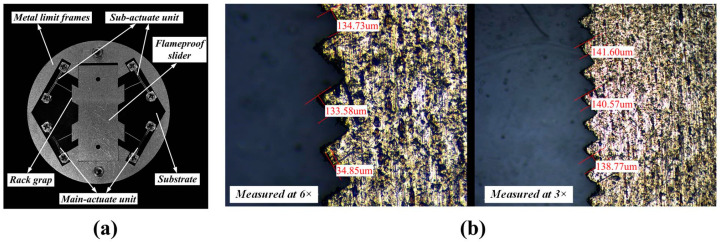
The prototype and detailed view of the multi-electrothermal co-actuation device: (**a**) the assembled prototype; and (**b**) the detailed view of the rack gap.

Based on the experimental equipment shown in Figure 19, an additional power supply is required for the sub-actuate unit in this experiment. The prototype needs to be assembled, as shown in Figure 23. Due to the small size of the components, the assembly process is carried out under a microscope. The electrothermal actuator with a pusher has holes punched at both ends of the anchor points, which are connected and fixed to the substrate using screws with corresponding positioning holes on the substrate, and a metal limit frame is used to limit the left and right movement of the flameproof slider, which is also fixed to the substrate through the positioning holes.

**Figure 24:**
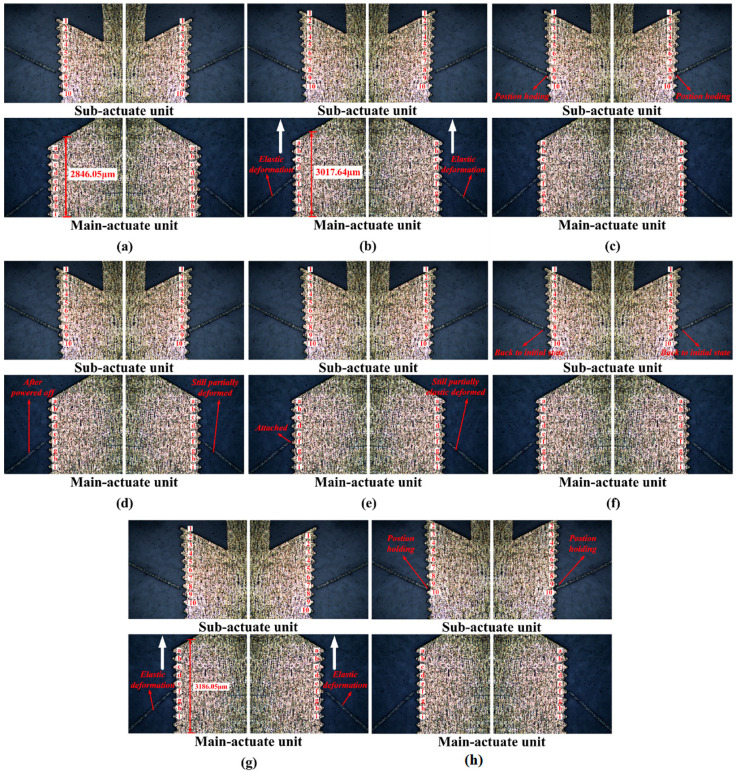
The working process and displacement output of the flameproof slider in a single cycle. (**a**) Initial state; (**b**) main-actuate unit pushes the slider to move one sub-step displacement; (**c**) sub-actuate unit holds the position of the slider; (**d**) main-actuate unit is powered off; (**e**) main-actuate unit attaches the slider; (**f**) sub-actuate unit is powered off; (**g**) main-actuate unit pushes the slider to output another sub-step displacement; and (**h**) sub-actuate unit holds the position of the slider again.

However, the experiment is not an ideal environment, and there is an external load and friction that cause energy loss when the electrothermal actuator outputs a displacement. So, in this experiment, 1.2 V is the most suitable voltage for the main-actuate unit, while 0.5 V is the optimal voltage for the sub-actuate unit. Combined with the driving strategy, periodic electrical signals of corresponding amplitude are applied to the main- and sub-actuate units. Figure 24 shows the prototype’s working process within one cycle. To facilitate understanding of how the slider outputs a displacement of 2Δ*y* within one cycle, the teeth on both the sub-actuate unit and main-actuate unit are numbered as 1, 2, 3, ……, 10 and a, b, c, ……, i respectively.

The levers of actuators 1 and 2 will deform elastically when the main-actuate unit drives the slider to output a forward displacement. Since the deformation is within the material limitation, the force between the lever and the rack gap disappears when powered off, and the levers gradually recover their deformation. However, there is still a slight residual deformation. In realizing the position-holding function, the sub-actuate unit, the lever, and the rack gap can be effectively jammed but do not push the slider, and the force between them is minimal. Due to the symmetry of the mechanism structure, the movement in the *−y* direction is identical to that in the +*y* direction.

Since the output displacement of the flameproof slider is directly affected by the machining accuracy of the actuator’s lever and slider and the assembly accuracy, there will inevitably be some dimensional variations even for the same batch of processing. Therefore, multiple experimental prototypes of the co-actuation device are assembled in this experiment, and three sets of displacement tests are performed on each prototype. The experimental results are presented in Table 2.

The repeatability test for three repetitions of test data for each prototype is evaluated by the relative standard deviation, and the results in Table 2 show that the repeatability error is less than 1%. The results show that the displacement performance test has good repeatability within the allowable experimental error of the same prototype.

However, there is some difference in displacement between the different prototypes, which is caused by laser processing and assembly errors. Despite these discrepancies, all the tested prototypes can achieve a displacement of at least 300 μm, demonstrating excellent driving performance.

The repeatability test for three repetitions of test data for each prototype is evaluated by the relative standard deviation, and the results in Table 2 show that the repeatability errors are less than 1%, and the test repeatability is good.

For further optimization, stainless steel WET [26] can be considered for integrated fabrication, which reduces the impact of machining and assembly errors on the device’s output performance.

## 7. Conclusions

This paper investigates a bidirectional device based on multi-electrothermal co-actuation, where the V-shaped actuators were fabricated using stainless steel 304. Theoretical analysis, simulation analysis, and experimental validation were conducted on its performance, and the results were consistent. The main findings are listed below.

(1) A finite element 3D model of the co-actuation device was established to verify the correctness of the theoretical model in describing the temperature distribution and displacement characteristics of the V-shaped actuator, and the dynamic response characteristics of the actuator as well as the whole system were further investigated. The results show that the co-actuation device can output two steps in a single cycle, achieving a total displacement of about 300.14 μm, with a system response time of about 1.2 s.
(2) The effects of the geometric factors on the displacement of the V-shaped actuator were studied. The sensitive geometric factors under different input conditions (voltage or temperature) were obtained through comparative analysis. Based on these findings, the optimized final geometric parameters of the V-shaped electrothermal actuator were determined, providing valuable data references for the prototype fabrication.
(3) The correctness of the theoretical and simulation analyses was verified by testing the steady-state displacement performance, temperature distribution, and dynamic response characteristics. The cumulative displacement performance of the multi-electrothermal co-actuation device within a single cycle was also tested. The slider could complete two steps along the +*y* direction with a total displacement of about 340 μm.
(4) The output displacement results from multiple tests on the same prototype exhibited good repeatability. There were some errors between the test results of the different prototypes. However, all the prototypes still met the driving requirements, which validated the rationality of the mechanism design and the feasibility for application in the fuze safety system.

## Figures and Tables

**Table 1:** The geometric parameters of the single V-shaped electrothermal actuator.

Parameter Name	Physical Unit	Specific Value
*L_z_*	μm	7000
*n*	μm	2
*d*	μm	200
*a*	μm	100
*S*	μm	200
*θ*	*°*	2
*t_v_*	μm	100
*L_p_*	μm	5000

where *S* is the gap between the V-beams, *n* is the number of V-beams, and *L_p_* is the effective length of the lever.

**Table 2:** The experimental results of displacements in a single cycle for different prototypes.

Prototype Number	Number of Tests			Average	RSD
	First Test	Second Test	Third Test		
Prototype 1	340 μm	337.31 μm	335.26 μm	337.523 μm	0.704%
Prototype 2	323.45 μm	320.56 μm	317.98 μm	320.663 μm	0.853%
Prototype 3	311.43 μm	309.45 μm	307.97 μm	309.617 μm	0.561%

## Data Availability

All data are presented within the paper.

## Abbreviations

The following abbreviations are used in this manuscript:

MEMS	Micro-Electromechanical Systems
PSO	Particle Swarm Optimization
WET	Wet-Etching Technology
RSD	Relative Standard Deviation
